# Metabolomics with severity of radiographic knee osteoarthritis and early phase synovitis in middle-aged women from the Iwaki Health Promotion Project: a cross-sectional study

**DOI:** 10.1186/s13075-022-02830-w

**Published:** 2022-06-17

**Authors:** Eiji Sasaki, Hiroyuki Yamamoto, Toru Asari, Rira Matsuta, Seiya Ota, Yuka Kimura, Shizuka Sasaki, Kyota Ishibashi, Yuji Yamamoto, Kenjiro Kami, Masataka Ando, Eiichi Tsuda, Yasuyuki Ishibashi

**Affiliations:** 1 Department of Orthopaedic Surgery, Hirosaki University Graduate School of Medicine, 5 Zaifu-cho, Hirosaki, Aomori, 036-8562 Japan; 2 Human Metabolome Technologies, Tsuruoka, Japan; 3 Department of Metabolomics Innovation, Hirosaki, Japan; 4 School of Allied Health Sciences, Kitasato University, Kanagawa, Japan; 5 Department of Rehabilitation Medicine, Hirosaki University Graduate School of Medicine, Hirosaki, Japan

**Keywords:** Knee osteoarthritis, Metabolites, Metabolome analysis, Radiographic osteoarthritis, Synovitis, Cohort study

## Abstract

**Background:**

Osteoarthritis (OA) is one of the costliest and most disabling forms of arthritis, and it poses a major public health burden; however, its detailed etiology, pathophysiology, and metabolism remain unclear. Therefore, the purpose of this study was to investigate the key plasma metabolites and metabolic pathways, especially focusing on radiographic OA severity and synovitis, from a large sample cohort study.

**Methods:**

We recruited 596 female volunteers who participated in the Iwaki Health Promotion Project in 2017. Standing anterior-posterior radiographs of the knee were classified by the Kellgren-Lawrence (KL) grade. Radiographic OA was defined as a KL grade of ≥ 2. Individual effusion-synovitis was scored according to the Whole-Organ Magnetic Resonance Imaging Scoring System. Blood samples were collected, and metabolites were extracted from the plasma. Metabolome analysis was performed using capillary electrophoresis time-of-flight mass spectrometry. To investigate the relationships among metabolites, the KL grade, and effusion-synovitis scores, partial least squares with rank order of groups (PLS-ROG) analyses were performed.

**Results:**

Among the 82 metabolites examined in this assay, PLS-ROG analysis identified 42 metabolites that correlated with OA severity. A subsequent metabolite set enrichment analysis using the significant metabolites showed the urea cycle and tricarboxylic acid cycle as key metabolic pathways. Moreover, further PLS-ROG analysis identified cystine (*p* = 0.009), uric acid (*p* = 0.024), and tyrosine (*p* = 0.048) as common metabolites associated with both OA severity and effusion-synovitis. Receiver operating characteristic analyses showed that cystine levels were moderately associated with radiographic OA (*p* < 0.001, area under the curve 0.714, odds ratio 3.7).

**Conclusion:**

Large sample metabolome analyses revealed that cystine, an amino acid associated with antioxidant activity and glutamate homeostasis, might be a potential metabolic biomarker for radiographic osteoarthritis and early phase synovitis.

**Supplementary Information:**

The online version contains supplementary material available at 10.1186/s13075-022-02830-w.

## Introduction

Osteoarthritis (OA), an age-related low-grade inflammatory disease of the synovial joints [1–3], is one of the costliest and most disabling forms of arthritis, being highly prevalent and posing a major public health burden [4, 5]. Although prevention and an early diagnosis are prerequisites for a favorable outcome, the detailed etiology, pathophysiology, and metabolism of OA remain unclear [6].

Recently, there has been increased interest in the early stages of knee OA [7, 8]. In this condition, the synovial joints present with various minute pathological changes in the cartilage, ligaments, menisci, subchondral bone, and synovium of the knee, even without radiographic abnormalities [9]. A longitudinal large sample cohort study revealed that bone marrow lesions, effusion-synovitis, and meniscal lesions are key pathologies of OA progression [10]. Additional epidemiological studies using magnetic resonance imaging (MRI) have reported that mild-to-moderate inflammation is detectable in people with early-stage knee OA [3, 11]. Pathophysiologically, synovitis has a strong impact on joint degeneration because the inflamed synovium secretes a variety of proteases and cytokines [12]. However, the precise biological and biomechanical mechanisms of OA are not fully understood yet.

Recent omics profiling approaches combined with clinical measures can comprehensively assess the health status and identify deviations from healthy baselines; these approaches may improve disease risk prediction and early detection [13]. Metabolomics detects many small-molecule metabolites in body fluids or tissues and quantifies these molecules in a single step. This approach promises an immense potential for early diagnosis, therapy monitoring, and understanding of the pathogenesis of many diseases [14]. However, previous omics analyses were affected by limitations in the sample size owing to the high running costs. Large sample cohort studies are necessary to analyze the severity-related changes and assess the influence of confounders like anthropometric factors, bone strength, hormonal condition, or synovitis. After recent improvements in analytical techniques had made it possible to conduct large sample metabolome analyses [15], several large-scale cohort studies successfully identified the etiologies and biomarkers in various fields of health maintenance [16, 17].

We employed targeted plasma metabolome analyses in this large sample cohort study to identify the metabolites and metabolic pathways associated with radiographic severity in OA of the knee. Additionally, we investigated the metabolic pathways related to synovitis and effusion to better understand the etiology of early-stage knee OA and identify the risk factors for the progression of knee OA. We expected that a novel inflammation-related biomarker associated with both synovitis and radiographic severity of knee OA would be identified and that the underlying metabolic pathways related to the etiology of early-stage knee OA would be revealed.

## Materials and methods

This was a cross-sectional study with volunteer participants from the Iwaki Health Promotion Project. The Iwaki Health Promotion Project, an annual program for the general population living in the Iwaki area of Hirosaki City located in the western Aomori prefecture, is a preventive medicine program that aims at improving average life expectancy by performing general health checkups [3, 8, 18]. The study of knee OA began in 2008, with annual checkups that followed the natural progression of the condition. At each checkup, physical examination, functional tests, laboratory findings, basic lifestyle information, and imaging data were registered. Furthermore, to discover the disease in its early phase, we performed MRIs on middle-aged female patients, who are known to have the highest risk for knee OA and its progression. The participants were community-dwelling residents who were recruited using mass media advertisements and with the help of public health nurses. All participants provided written informed consent. The study was conducted in agreement with the 1964 Helsinki Declaration and its later amendments or comparable ethical standards and was approved by the ethics committee of Hirosaki University Graduate School of Medicine, Japan (2017-026).

### Participants

**Fig. 1:**
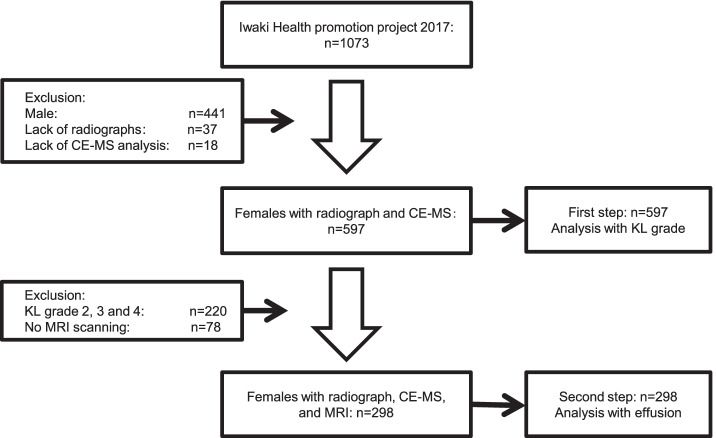
Flowchart of the participant enrollment process. CE-MS, capillary electrophoresis mass spectrometry; KL, Kellgren-Lawrence; MRI, magnetic resonance imaging

Out of 10,000 community-dwelling residents, 1073 volunteers who participated in the Iwaki Health Promotion Project 2017 were enrolled in this study. First, participants who had not undergone radiographic and blood examinations were excluded. This study aimed to identify the biomarkers useful for the detection of early and advanced stages of OA. Two phases were conducted. Phase 1 analysis aimed to discover the metabolites related to increased radiographic severity in high-risk female participants, who are the definitive high-risk group for knee OA. Therefore, 441 men were excluded, and a total of 597 women were assessed (Fig. 1). In phase 2, we focused on the synovitis without radiographic abnormality, in order to assess the discovered metabolites in the early phase of knee OA. MRIs in phase 2 were performed only on middle-aged women who are at a high risk of early knee OA [8], resulting in a total of 298 participants.

### Blood sampling and metabolite extraction

Blood samples were collected from all participants before breakfast after a period of 10 or more hours of fasting. After centrifugation, collected plasma were separately stored at – 80 °C. Metabolite extraction and metabolome analysis were conducted at Human Metabolome Technologies (HMT, Tsuruoka, Yamagata, Japan). Briefly, 50 μL of plasma was added to 450 μL of methanol containing internal standards (Solution ID: H3304-1002, HMT, Inc., Tsuruoka, Japan) at 0 °C to inactivate enzymes. The extract solution was thoroughly mixed with 500 μL of chloroform and 200 μL of Milli-Q water and centrifuged at 2300×*g* and 4 °C for 5 min; 350 μL of the upper aqueous layer was centrifugally filtered through a Millipore 5-kDa cutoff filter to remove the proteins. The filtrate was centrifugated, the pellet resuspended in 50 μL of Milli-Q water, and then used for metabolome analysis at the HMT.

### Metabolome analysis

Metabolome analysis was conducted with the Basic Scan package of the HMT using capillary electrophoresis time-of-flight mass spectrometry (CE-TOFMS) based on the methods described previously [19, 20]. Briefly, CE-TOFMS analysis was carried out using an Agilent CE capillary electrophoresis system equipped with an Agilent 6210 time-of-flight mass spectrometer, Agilent 1100 isocratic HPLC pump for sheath liquid, Agilent G1603A CE-MS adapter kit, and Agilent G1607A CE-ESI-MS sprayer kit (Agilent Technologies, Waldbronn, Germany). The systems were controlled by the Agilent G2201AA ChemStation software version B.03.01 for CE (Agilent Technologies) and connected by a fused silica capillary (50 μm inner diameter × 80 cm total length) with a commercial electrophoresis buffer (H3301-1001 and H3302-1021 for cation and anion analyses, respectively; HMT) as the electrolyte. The spectrometer was scanned from *m*/*z* 50 to 1000 [19]. Peaks were extracted using MasterHands, an automatic integration software (Keio University, Tsuruoka, Yamagata, Japan), to obtain peak information including m/z, peak area, and migration time [21]. Signal peaks corresponding to isotopomers, adduct ions, and other product ions of known metabolites were excluded, and the remaining peaks were annotated according to the HMT metabolite database based on their *m*/*z* values with the migration times. Areas of the annotated peaks were then normalized based on internal standard levels and sample volumes to obtain relative levels of each metabolite. The relative levels were further normalized using control plasma samples (Sigma-Aldrich Japan, Tokyo, Japan) when measurement conditions were changed by different analysis equipment or capillary replacement in CE-MS measurement. The coefficients of variation (CVs) reported in our previous study were calculated using a portion of the same sample as in this study [22]. The minimum and maximum values of CVs were 3.3% and 81.6%, respectively. For the detected metabolites, 61.6% of the number of metabolites had CVs less than 10%. Moreover, on the basis of the metabolomic results analysis, plasma cystine concentrations (nmol/mL, HPLC) were quantified by the LSI Medience Corporation (LSI Medience Corp., Tokyo, Japan).

### Radiographic evaluation

Radiographic examinations of the knee were performed using a digital radiographic device (CXDI-40EG, Canon Inc. Tokyo, Japan). Experienced radiologists and orthopedic surgeons obtained weight-bearing, full-extension, and anterior-posterior radiographs of both the knees with foot map positioning on the day of the checkup. The beam was positioned parallel to the floor and aimed at the joint space, and the sequencing was set at 60 kV, 50 mAs, and 80 ms for all participants. The images were converted into Joint Photographic Experts Group format files. OA severity in each knee was classified by two trained orthopedic surgeons (D.C. and S.O.) as KL grades 0–4 using the KL radiographic atlas [23]. The interclass correlation coefficient of the assessment performed by the two surgeons was 0.815, and the intraclass correlation coefficient was 0.923. These surgeons were unaware of the sequence in which the radiographs were acquired and the clinical status of the participants.

### Synovitis evaluated using MRI

Synovitis on MRI was assessed in the right knee. Participants were positioned in supine with their knees in full extension and scanned with a rapid extremity coil and mobile magnetic resonance unit (1.5 T; ECHELON RX, Hitachi, Tokyo, Japan) within 1 week of the other examinations. The scanning sequences were set including the sagittal and coronal planes of T2-weighted fat-suppressed fast-spin echo sequences (repetition time 5000 ms; echo time 80 ms; field of view 16 cm; 288 × 288 matrix; slice thickness of 3 mm with a gap of 1.0 mm between slices). The image database was transferred to an independent computer workstation using the OsiriX software (Newton Graphic, Inc., Hokkaido, Japan). According to the Whole-Organ MRI Scoring (WORMS) method [24], synovitis was semi-quantitatively graded as an effusion-synovitis score from 0 to 3 in terms of the estimated maximal distention of the synovial cavity: 0 = normal, 1 = < 33% of maximum potential distention, 2 = 33–66% of maximum potential distention, and 3 = > 66% of maximum potential distention. Based on the effusion-synovitis scores, participants with KL grades 0 and 1 were divided into low (effusion-synovitis score of 0; *n* = 205) and high (effusion-synovitis score of 1, 2, and 3; *n* = 93) effusion-synovitis groups to determine the absence or presence of synovitis, respectively. Furthermore, the effusion-synovitis volume (cm^3^) was quantified at the suprapatellar fluid equivalent signal on MRI using the OsiriX software by an expert orthopedic surgeon (S.O.). To validate the measurements of effusion synovitis volume, the intra-class correlation coefficient (ICC) was estimated from two separate measurements. The ICC (1,1) of the effusion-synovitis volume was 0.984.

### Covariates

The patients’ anthropometric parameters including height, weight, and body mass index (BMI) values were recorded. The difference in muscle volume (kg) of participants was estimated by measuring impedance with the Tanita MC-190 body composition analyzer (Tanita Co., Ltd., Tokyo, Japan), since muscular volume is correlated to the concentration of some metabolites. In this study, the muscle volume was evaluated as that of the right lower extremity, including the thigh, in order to correspond to the side of the obtained MRI.

Furthermore, the serum inflammatory biomarkers were measured from the collected blood samples. After centrifugation, the collected sera were separately stored at – 80 °C. Serum samples were analyzed by the LSI Medience Corporation (LSI Medience Corp., Tokyo, Japan). The company has an ISO-15189-accredited laboratory; therefore, serum assays were performed under strict conditions. As biomarkers for inflammation, highly sensitive C-reactive protein (mg/dL, Nephelometry), matrix metalloproteinase 3 (mg/mL, LA), interleukin-6 (pg/mL, CLEIA), and hyaluronic acid (ng/mL, LA) levels were assessed. The reliabilities of these biomarkers, as shown in the coefficient of variation, were 1.0–1.5%, 2.0–4.8%, 1.5–7.4%, and 2.3–5.0%, respectively.

### Statistical analysis

Demographic data and serum inflammation markers are presented as mean ± standard deviation. Age, BMI, muscular volume, and serum inflammation biomarkers among KL grades were compared by the analysis of variance. In the phase 1 analysis, partial least squares (PLS) and PLS with rank order of groups (PLS-ROG) were used. While PLS considers only group differences, PLS-ROG considers the group differences as well as information about the order of the groups. Therefore, PLS-ROG is useful when, in addition to information on the groups, information on the order of groups, such as the KL grade, is available. We first performed PLS and confirmed the association between PLS scores and KL grades. If there was no association between the PLS scores and KL grades, we performed PLS-ROG analyses. Statistically significant metabolites related to the KL grade were identified using PLS or PLS-ROG loading [25] based on Pearson’s correlation coefficient (*r*) between the PLS-ROG score for the response variable and each metabolite level. We set the smoothing parameter *κ* as 0.999 in the PLS-ROG analyses. A previous study that explained the theoretical aspects of PLS-ROG [25] reported how the score changes when the smoothing parameter is varied. For practical purposes, it is sufficient to set the smoothing parameter close to 1 (0.999 in this study) and to confirm whether the scores are associated with the order of the groups by visual inspection. Using the identified metabolites, metabolite set enrichment analyses were performed to detect the related pathways by over-representation analysis [26] using an HMT proprietary program written in R [27] and the same metabolite setlist used in our previous study [27]. A heatmap and dendrograms of metabolites were created based on the metabolites’ intensities. In this analysis, *Z*-scores of the detected metabolites are shown in each row, and samples that were sorted with the Kellgren-Lawrence grade are shown in each column.

In the phase 2 analysis, levels of metabolites between low and high effusion-synovitis score groups were compared using the Mann-Whitney *U* test. Furthermore, PLS-ROG analysis of the effusion-synovitis score and metabolites was performed, and related metabolites were identified using PLS-ROG loading. Then, Spearman’s correlation coefficients (*ρ*) were estimated to investigate the relationship between quantified plasma cystine consentrations and inflammation markers.

Finally, to estimate the cutoff value for detecting knee OA with KL grade 2 and higher, a receiver operating characteristic (ROC) curve analysis was performed using these commonly correlated metabolites. Based on the calculated areas under the curve (AUC), the strength with the presence of OA was compared among the metabolites, and their cutoff values were defined as the nearest point to the true positive, that is, the left upper corner of the plot box to maximize the true positive fraction and minimize the false-positive fraction.

Calculations for the statistical analysis were performed using the HMT proprietary R program, SPSS version 25.0J (SPSS Inc., Chicago, IL, USA), and the statistical software BellCurve for Excel (version 3.21; Social Survey Research Information Co., Ltd., Tokyo, Japan). A *p*-value and *q*-value, for multiple comparison testing based on the Benjamini-Hochberg [28] approach, < 0.05 were considered statistically significant.

## Results

**Fig. 2:**
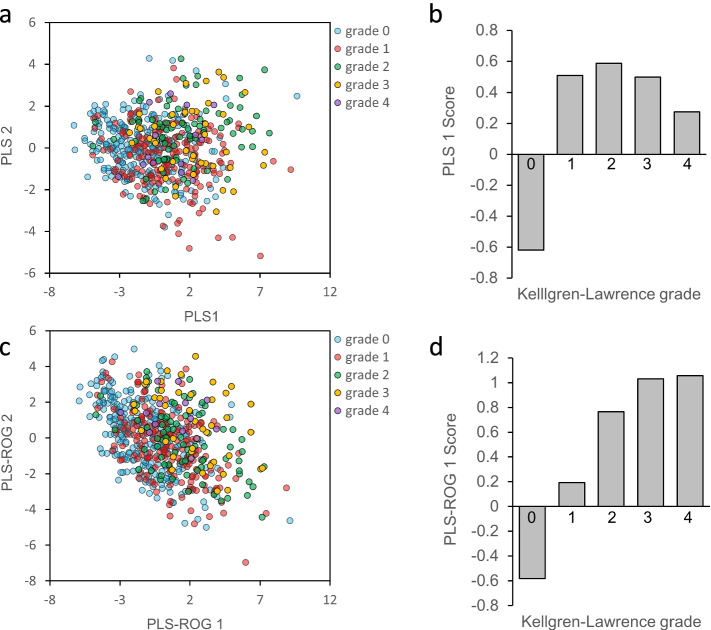
PLS and PLS-ROG analyses of the Kellgren-Lawrence (KL) grade and metabolites. Results of the partial least squares (PLS) and PLS with rank order of groups (PLS-ROG) analyses of the metabolome data. The KL grade (0–4) was set as the response variable. A scatter plot of the first and second PLS scores for the explanatory variable (**a**). A bar plot (**b**) of the first PLS score for the response variable and a scatter plot (**c**) of the first and second PLS-ROG score for the explanatory variable, are shown. A bar plot (**d**) of the first PLS-ROG score for the response variable is shown

Among the 597 women, a participant who was an outlier, exceeding the 99% confidence interval in a principal component analysis score plot, was excluded (Fig. S1) [27]. Finally, the initial phase 1 analysis included 596 female participants (Fig. 1); their demographic and clinical data are shown in Table 1. In total, 82 metabolites were examined and shown in a heatmap (Fig. S2 and Additional file 1: Table S1). We performed PLS analyses (Fig. 2a, b) of the metabolome data, which failed to confirm an association of PLS scores with OA severity based on the KL grade. Therefore, PLS-ROG analysis for the association between the KL grade and metabolites was performed (Fig. 2c, d). This analysis confirmed an association of the first PLS-ROG score with the KL grade. Of the 82 metabolites, 37 were positively and five were negatively correlated with radiographic severity (*q* < 0.05). The correlated metabolites (from the highest correlation downwards) were cystine (*r* = 0.397, *p* < 0.001, *q* < 0.001), urea (*r* = 0.392, *p* < 0.001, *q* < 0.001), *cis*-aconitic acid (*r* = 0.290, *p* < 0.001, *q* < 0.001), ornithine (*r* = 0.254, *p* < 0.001, *q* < 0.001), and tyrosine (*r* = 0.214, *p* < 0.001, *q* < 0.001). A subsequent metabolite set enrichment analysis using the significant metabolites (*p* < 0.05) found no significantly associated metabolic pathway (Additional file 1: Table S2), whereas five out of six metabolites in the urea cycle (*p* = 0.059, *q* =1.000) and all the three metabolites in the tricarboxylic acid (TCA) cycle (*p* = 0.085, *q* =1.000) were identified as key metabolites.

**Table 1:** Demographic and clinical data of participants according to their Kellgren-Lawrence grade

	KL 0	KL 1	KL 2	KL 3	KL 4	*p*-value
Number of participants	271	187	79	45	14	
Age (years)	45.9 ± 13.6	57.3 ± 11.6	66.0 ± 11.4	71.9 ± 7.5	69.6 ± 9.0	< 0.001
BMI (kg/m^2^)	21.2 ± 3.2	22.8 ± 3.5	23.3 ± 3.8	25.3 ± 4.3	25.5 ± 5.4	< 0.001
Muscle volume (kg)	6.3 ± 0.7	6.2 ± 0.9	5.9 ± 0.8	5.9 ± 0.7	6.0 ± 0.6	< 0.001
hs-CRP (mg/dL)	0.06 ± 0.16	0.06 ± 0.13	0.06 ± 0.13	0.08 ± 0.16	0.06 ± 0.11	0.918
MMP-3 (mg/mL)	36.4 ± 18.7	37.3 ± 14.5	34.0 ± 11.9	38.1 ± 13.1	42.6 ± 12.5	0.319
IL-6 (pg/mL)	1.8 ± 6.3	1.0 ± 1.2	1.2 ± 1.1	1.2 ± 0.7	0.7 ± 0.4	0.408
HA (ng/mL)	48.6 ± 64.7	46.8 ± 49.7	45.5 ± 50.4	80.3 ± 86.0	40.7 ± 93.2	0.016

Data are presented as the mean ± standard deviation and were compared using analysis of variance tests. The muscle volume refers to that of the right thigh and lower leg

*BMI* body mass index, *HA* hyaluronic acid, *hs-CRP* high-sensitive C-reactive protein, *IL-6* interleukin-6, *KL* Kellgren-Lawrence grade, *MMP-3* matrix metalloproteinase 3

**Fig. 3:**
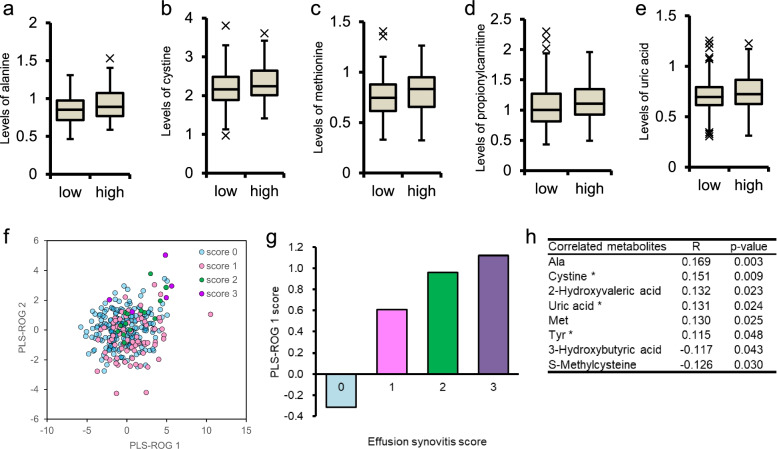
Effusion-synovitis-related metabolites. Levels of metabolites between the low (effusion-synovitis score 0) and high (effusion-synovitis scores 1, 2, and 3) groups are compared by the Mann-Whitney *U* test. Levels of alanine (**a**), cystine (**b**), methionine (**c**), propionylcarnitine (**d**), and uric acid (**e**) in the high group are significantly higher than those of the lower group. The box and center line indicate the first quartile, median, and third quartile. The dots indicate the outliers. Partial least squares with rank order of group (PLS-ROG) results for metabolome data (**e–g**). The effusion-synovitis score (from 0 to 3) was set as the response variable. A scatter plot of the first and second PLS-ROG scores for the explanatory variable (**f**). A bar plot of the first PLS-ROG score for the response variable (**g**). Significant metabolites in the PLS-ROG analysis with effusion-synovitis scores (**h**). Metabolites that correlate with both the Kellgren-Lawrence grade and effusion-synovitis score are indicated with an asterisk (*)

The phase 2 analysis included 298 women who had undergone MRI examinations. In this population, the mean effusion-synovitis volume was 2.6 ± 2.7 (range 0.1–24.6) cm^3^, which increased with the effusion-synovitis score (*p* < 0.001; Additional file 1: Table S3). There were no significant differences in age, BMI, and muscle volume in the effusion-synovitis score in the variance test analysis (Additional file 1: Table S3). In the comparison between the low and high effusion-synovitis score groups, alanine (*p* = 0.016, *q* = 0.587), cystine (*p* = 0.039, *q* = 0.587), methionine (*p* = 0.043, *q* = 0.587), propionylcarnitine (*p* = 0.046, *q* = 0.587), and uric acid (*p* = 0.048, *q* = 0.587) were identified as metabolites with significantly increased levels in patients with high synovitis scores (*p* < 0.05; Fig. 3a–e).

**Fig. 4:**
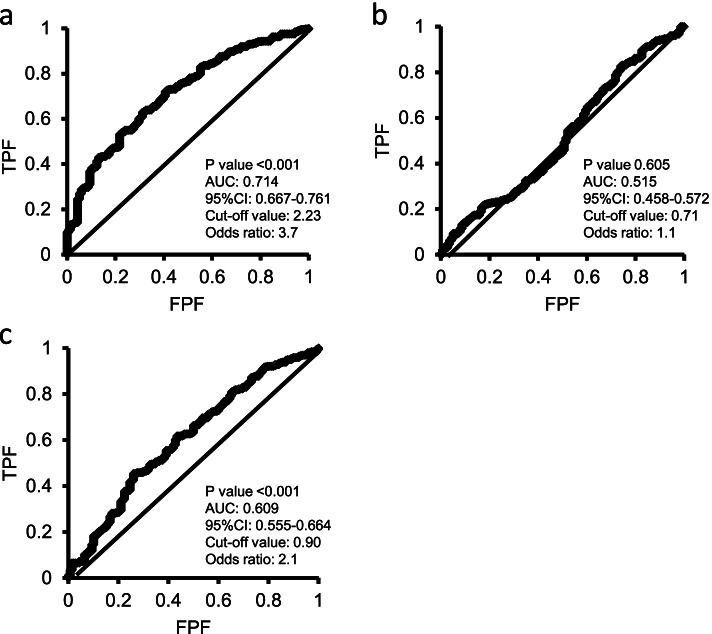
Receiver operating characteristic (ROC) analysis of metabolites for the presence of radiographic knee osteoarthritis. ROC analysis was performed to estimate the predictivity of cystine (**a**), uric acid (**b**), and tyrosine (**c**) for the presence of radiographic knee osteoarthritis. Cystine has the highest area under the curve value with a cutoff value of 2.23 and an odds ratio of 3.7. TPF, true-positive fraction; FPF, false-positive fraction

Regarding the metabolomics results related to effusion-synovitis scores in patients with KL grades 0 and 1, the PLS-ROG analysis detected eight significantly correlated metabolites, which were alanine, cystine, 2-hydroxyvaleric acid, uric acid, methionine, tyrosine, 3-hydroxybutyric acid, and *S*-methylcysteine (*p* < 0.05; Fig. 3f–h). Among these metabolites, cystine (*r* = 0.151, *p* = 0.009, *q* = 0.375), uric acid (*r* = 0.131, *p* = 0.024, *q* = 0.403), and tyrosine (*r* = 0.115, *p* = 0.048, *q* = 0.437) were identified as common key metabolites associated with both the KL grade and effusion-synovitis score (Fig. 3h). Further ROC analyses showed that the cystine level was moderately associated with the presence of radiographic OA (*p* < 0.001; AUC 0.714; 95% CI 0.667, 0.761; cutoff value 2.23; odds ratio 3.7; Fig. 4). The quantified plasma cystine consentration had a mean value of 28.4 ± 9.1 nmol/mL and was significantly correlated with the levels of serum inflammatory markers such as high-sensitive C-reactive protein (*ρ* = 0.252, *p* < 0.001), matrix metalloproteinase 3 (*ρ* = 0.137, *p* < 0.001), interleukin-6 (*ρ* = 0.224, *p* < 0.001), and hyaluronic acid (*ρ* = 0.467, *p* < 0.001).

## Discussion

This cross-sectional study accomplished a large sample metabolome analysis focusing on both radiographic knee OA and synovitis on MRI. Our results revealed that cystine, uric acid, and tyrosine were common metabolites related to both radiographic severity and degree of synovitis effusion. According to the ROC analysis, cystine highly reflected the presence of OA. High cystine levels may be a key biomarker in identifying patients at high risk of knee OA progression caused by synovitis. In addition, we demonstrated that a metabolomic approach could identify the key metabolites and metabolic pathways related to radiographic severity and effusion-synovitis, although the correlation was extremely weak.

Metabolomics has become an advanced method for the identification of OA biomarkers and can be used to systematically study small-molecule metabolites across biological systems. The metabolome constitutes the final downstream product of distinct regulatory complexes such as the genome, transcriptome, and proteome that are proximal to the metabolome constituting the disease phenotype [29]. Synovial fluid is an ideal specimen for OA studies because metabolite concentrations in the synovial fluid directly reflect the biological processes of the synovium or cartilage. However, obtaining synovial fluid samples is invasive and not practical for patients without joint effusion. Thus, one of the most frequently used biological samples in OA research is blood. Zhang et al. reported that correlations between blood plasma and synovial fluid metabolite concentrations are modest. Metabolite ratios, which are considered proxies for enzymatic reaction rates owing to their high correlation, should be considered when using blood plasma as a surrogate for synovial fluid in OA biomarker identification, while there are possibilities of including different metabolites or concentrations [30].

**Fig. 5:**
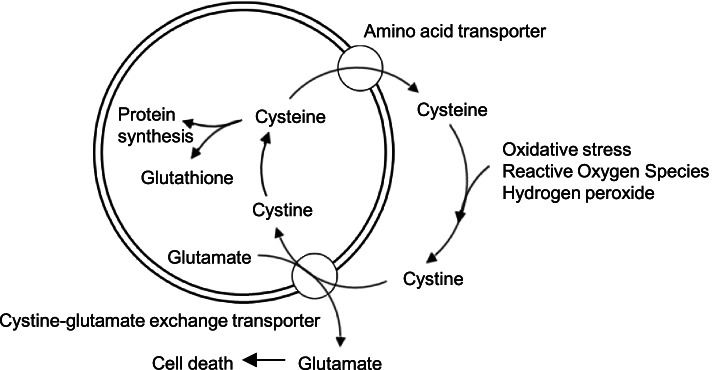
Schema of the cystine-cysteine cycle in knee osteoarthritis. Under excessive oxidative stress, cysteine is oxidized by hydrogen peroxide to cystine and transported into cells via the cystine-glutamate exchange transporter in the plasma membrane, and the transported cystine is intracellularly reduced to cysteine and used for the synthesis of proteins and glutathione. Glutathione has important roles in reducing reactive oxygen species. Increased extracellular cystine levels reflect the presence of oxidative stress in or around cells. Glutamate released via the cystine-glutamate transporter damages the chondrocytes, and high glutamate levels in the synovial fluid are accompanied by severe joint destruction in animal models of osteoarthritis. Although excessive oxidative stress can be controlled via the cystine-cysteine cycle, higher glutamate would also result in worse metabolic conditions for joint homeostasis

In our analyses, cystine was identified as a potential biomarker for identifying patients with both radiographic abnormality and active synovitis. Patients with active synovitis are considered a high-risk group for OA progression because synovitis leads to the secretion of several proteases and subsequent cartilage degeneration [1, 12]. Cystine is an amino acid formed from two molecules of the amino acid cysteine, which is well known as an anti-oxidative agent in the cystine-cysteine cycle [31]. Under excessive oxidative stress, cysteine is oxidized by hydrogen peroxide to cystine and transported into cells via the cystine-glutamate exchange transporter in the plasma membrane (Fig. 5) [31]. In macrophages, this transporter is also induced and activated by stimulation with reactive oxygen species [32]. The transported cystine is intracellularly reduced to cysteine and used for the synthesis of proteins and glutathione [33]. Glutathione has important roles in reducing reactive oxygen species [34]. Therefore, increased extracellular cystine levels reflect the presence of oxidative stress in or around cells. It is well known that oxidative stress causes inflammation of the synovium, thereby accelerating chondrocyte death, cartilage degeneration, and bone fragility [35]. Based on the described etiology and metabolism, cystine might be a novel biomarker for knee OA with synovitis.

Furthermore, glutamate released via the cystine-glutamate transporter is known to damage chondrocytes, and high glutamate levels in the synovial fluid were accompanied by severe joint destruction in an OA animal model [36]. In this model, inhibition of the cystine-glutamate transporter could prevent cartilage destruction [37]. Although excessive oxidative stress can be controlled via the cystine-cysteine cycle, higher glutamate would also result in worse metabolic conditions for joint homeostasis. From this perspective, an effective activation of the cystine-cysteine cycle without causing glutamate accumulation in the synovial fluid might be the key to reduce inflammation and the damage of chondrocytes and thereby and to alleviate the progression of the symptoms in knee OA.

Our metabolome analysis identified urea and TCA cycles as pathways related to knee OA severity; however, their correlation was very weak, and further studies on this would be needed. A previous targeted metabolome analysis showed that both cycles were key metabolic pathways for blood and synovial fluid metabolites [30]. The urea cycle, transferring nitrogen of an amino acid to the end metabolite urea in the mitochondria, is necessary to reduce ammonia toxicity. In progressed stages of OA, higher levels of synovitis are present [38], requiring more energy. To maintain the energy balance, for example, collagen and amino acid breakdown and TCA cycle might be upregulated, leading to the release of excessive amounts of amino groups. These amino groups are usually converted to ammonia, which also exerts toxicity. To reduce the excess amino group, the urea cycle might be activated. In our study, arginine levels in the KL grade 4 group were lower than those in the KL grade 0–3 group. Thus, our results showed that arginine was depleted in patients with progressed OA, similar to the findings in a previous report [39]. In a targeted metabolomics analysis. Zhang et al. found that depletion of plasma arginine is highly correlated with knee OA progression and might be decreased owing to its metabolic consumption [39]. Arginine is a semi-essential amino acid, a collagen precursor, and a natural inhibitor of cathepsins, which is considered as an important factor of OA pathogenesis [40].

Previous reports have suggested that the TCA cycle is one of the major pathways in immunometabolism [41]. The mitochondria are powerhouses of the cell, providing energy in the form of adenosine triphosphate for a range of activities including movement, cellular differentiation, cell death, regulation of signaling, and cell cycle control [42]. However, mitochondrial dysfunction and oxidative stress are hallmarks of OA [43], and increased mitochondrial DNA damage can be detected in chondrocytes from patients with OA compared with chondrocytes from healthy individuals [44]. Degeneration and inflammation sometimes limit the pyruvate metabolism in the mitochondrial TCA cycle during oxidative phosphorylation in the articular cartilage, subchondral bone, and synovium in patients with OA owing to the activity of synovial macrophages, even under aerobic conditions [45], and this may cause a stagnation of the TCA cycle, leading to a gradual accumulation of TCA intermediates in the blood in patients with high KL grades.

This study has several limitations. First, the biomarker candidates are limited to highly polar ionic metabolites because the metabolome analysis was performed using only CE-MS. Second, dietary intake, which can influence the levels of plasma metabolites, was not recorded in detail [46]. To reduce the risk of bias in our study, plasma was sampled after at least 10 h of fasting. Third, all the participants were women, which poses a risk of selection bias. The etiology of OA in men and women seems to be partially different [4]. The reduction of estrogen concentration with menopause is essential, and it influences the chondrocytes and cartilage [47, 48]. The influence of menopause should be assessed in future studies. In this study, we focused on middle-aged and older adult women because the highest prevalence of early-stage knee OA was observed in this age bracket [7]. For this reason, our results may not be completely applicable to men. Furthermore, the relationship with synovitis through all KL grades was not investigated in this cohort. Fourth, knee symptoms and functional tests were not included in this study. Patient-reported outcomes and functional scores reflect synovitis, knee symptoms, and severity of knee OA; the analysis of these data would have supplied important additional information. Fifth, in the ROC analysis, the AUC of cystine was below 0.8, which represents a fair correlation with the presence of radiographic OA. This reflects the difficulty in explaining the multifactorial etiology of this disease with one metabolite. In addition, the use of inflammatory markers and serum or plasma measurements has a risk of selection bias in this analysis. Sixth, while radiographs are very sensitive, they may not be appropriate for defining the stages of diseases because their reproducibility is considered to be low. Therefore, we added MRI evaluations to radiographic evaluations to investigate synovitis. Additionally, due to the high cost and time consumption of MRIs, they were only performed on the right knees. Finally, this was a cross-sectional study that used data collected in a cohort study; further studies with a longitudinal design are needed to reveal the causal relationships between these metabolic markers and OA.

## Conclusions

In summary, large sample metabolome analyses revealed that, in women, the plasma cystine level increased with an increase in both radiographic OA severity and MRI-based synovitis stage. Thus, cystine level is a potential biomarker for patients who have knee OA with active synovitis. In contrast, the urea and TCA cycles were considered as possible key metabolic pathways related to the severity of radiographic knee OA, although the association was very weak. Further longitudinal observations might reveal the prognostic power of the identified metabolites.

## Supplementary Information

**Additional file 1: Figure S1.** Principal component analysis plot of metabolites with Kellgren-Lawrence grade. The 99.999% confidence interval of each component is shown as a circle around the dot plot. An outlier was omitted for the statistical analysis. **Figure S2.** Heatmap of metabolites in relation with Kellgren-Lawrence grade. Z-scores of detected metabolites are presented in each row, and samples that were sorted with the Kellgren–Lawrence grade are presented in each column. **Table S1.** Results of the first PLS-ROG loading in correlation with the Kellgren-Lawrence grade. **Table S2.** Metabolite set enrichment analysis to detect metabolic pathway with severity of knee osteoarthritis. “All” includes the number of metabolites registered in metabolic pathway list. “Detected” includes the number of detected metabolites. “Selected” includes statistically significant metabolites with a p < 0.05. The p-value was computed by Fisher’s exact test and the q-value is its multiple comparison correction by Benjamini-Hochberg method. **Table S3.** Demographic data of participants according to their effusion-synovitis score. Data are presented as the mean ± standard deviation. Groups are compared by analysis of variance and Tukey test. BMI: body mass index.

## Data Availability

The original contributions presented in the study are included in the article/supplementary material. Further inquiries can be directed to the corresponding author.

## Abbreviations

BMI: Body mass index
OA: Osteoarthritis
PLS: Partial least square
PLS-ROG: PLS with rank order of groups
TCA: Tricarboxylic acid
ROC: Receiver operating characteristic
AUC: Area under the curve
KL: Kellgren-Lawrence
WORMS: Whole-Organ MRI Scoring
