# Supplementary material for: Metabolomics with severity of radiographic knee osteoarthritis and early phase synovitis in middle-aged women from the Iwaki Health Promotion Project: a cross-sectional study
Source: Arthritis Res Ther. 2022 Jun 17;24:145. doi: 10.1186/s13075-022-02830-w (PMC9205107; doi:10.1186/s13075-022-02830-w)
Supplement: Supplementary file 1 — Additional file 1: Figure S1. Principal component analysis plot of metabolites with Kellgren-Lawrence grade. The 99.999% confidence interval of each component is shown as a circle around the dot plot. An outlier was omitted for the statistical analysis. Figure S2. Heatmap of metabolites in relation with Kellgren-Lawrence grade. Z-scores of detected metabolites are presented in each row, and samples that were sorted with the Kellgren–Lawrence grade are presented in each column. Table S1. Results of the first PLS-ROG loading in correlation with the Kellgren-Lawrence grade. Table S2. Metabolite set enrichment analysis to detect metabolic pathway with severity of knee osteoarthritis. “All” includes the number of metabolites registered in metabolic pathway list. “Detected” includes the number of detected metabolites. “Selected” includes statistically significant metabolites with a p < 0.05. The p-value was computed by Fisher’s exact test and the q-value is its multiple comparison correction by Benjamini-Hochberg method. Table S3. Demographic data of participants according to their effusion-synovitis score. Data are presented as the mean ± standard deviation. Groups are compared by analysis of variance and Tukey test. BMI: body mass index. [file 13075_2022_2830_MOESM1_ESM.docx]

**
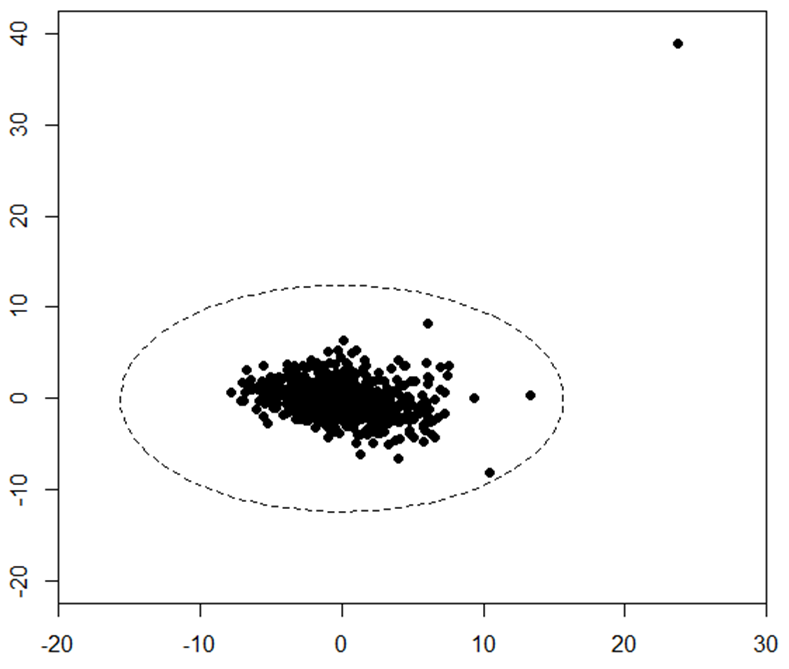
** **Supplementary Fig. 1. Principal component analysis plot of metabolites with Kellgren-Lawrence grade.** The 99.999% confidence interval of each component is shown as a circle around the dot plot. An outlier was omitted for the statistical analysis.

**
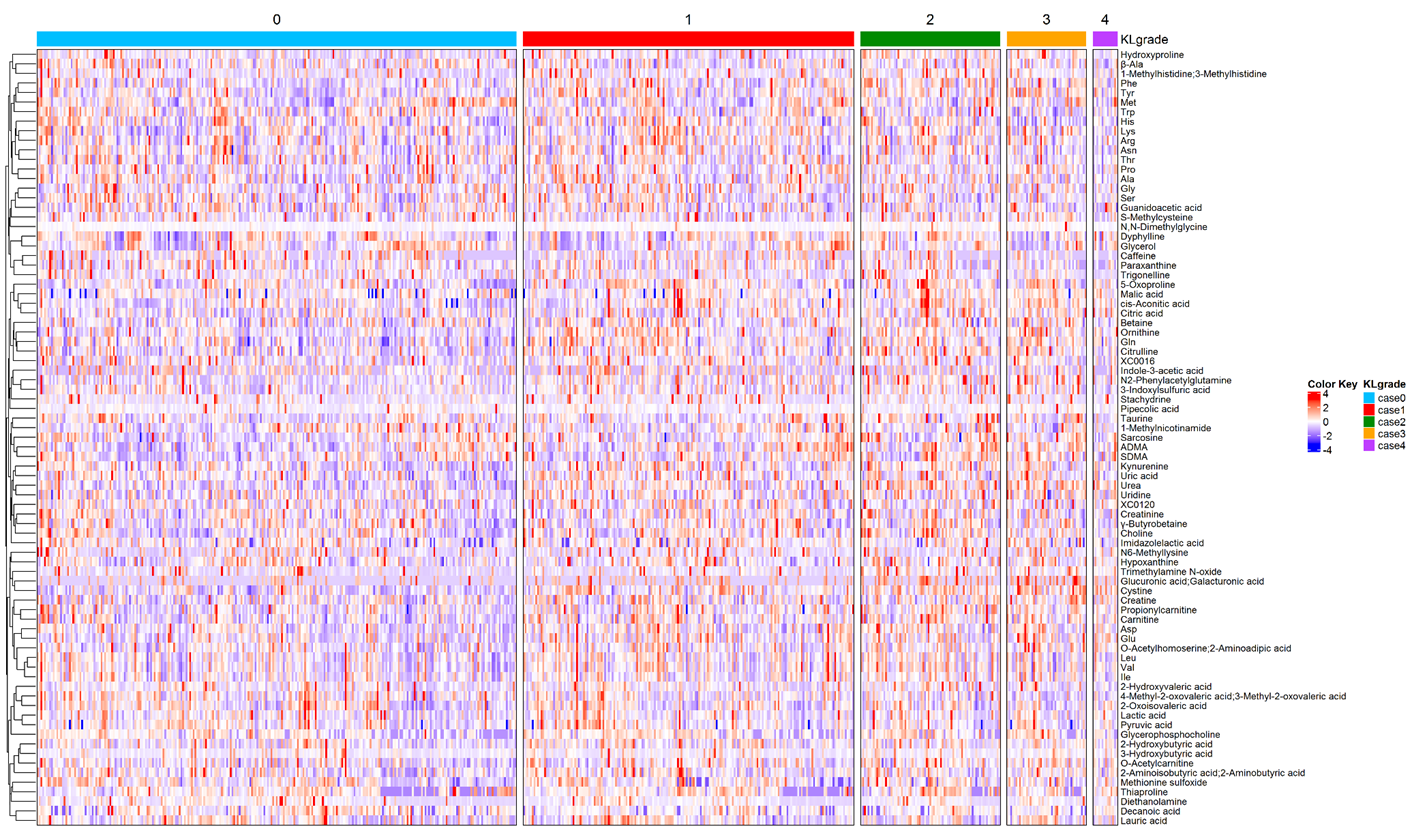
**

**Supplementary Fig. 2. Heatmap of metabolites in relation with Kellgren-Lawrence grade.** Z-scores of detected metabolites are presented in each row, and samples that were sorted with the Kellgren–Lawrence grade are presented in each column.


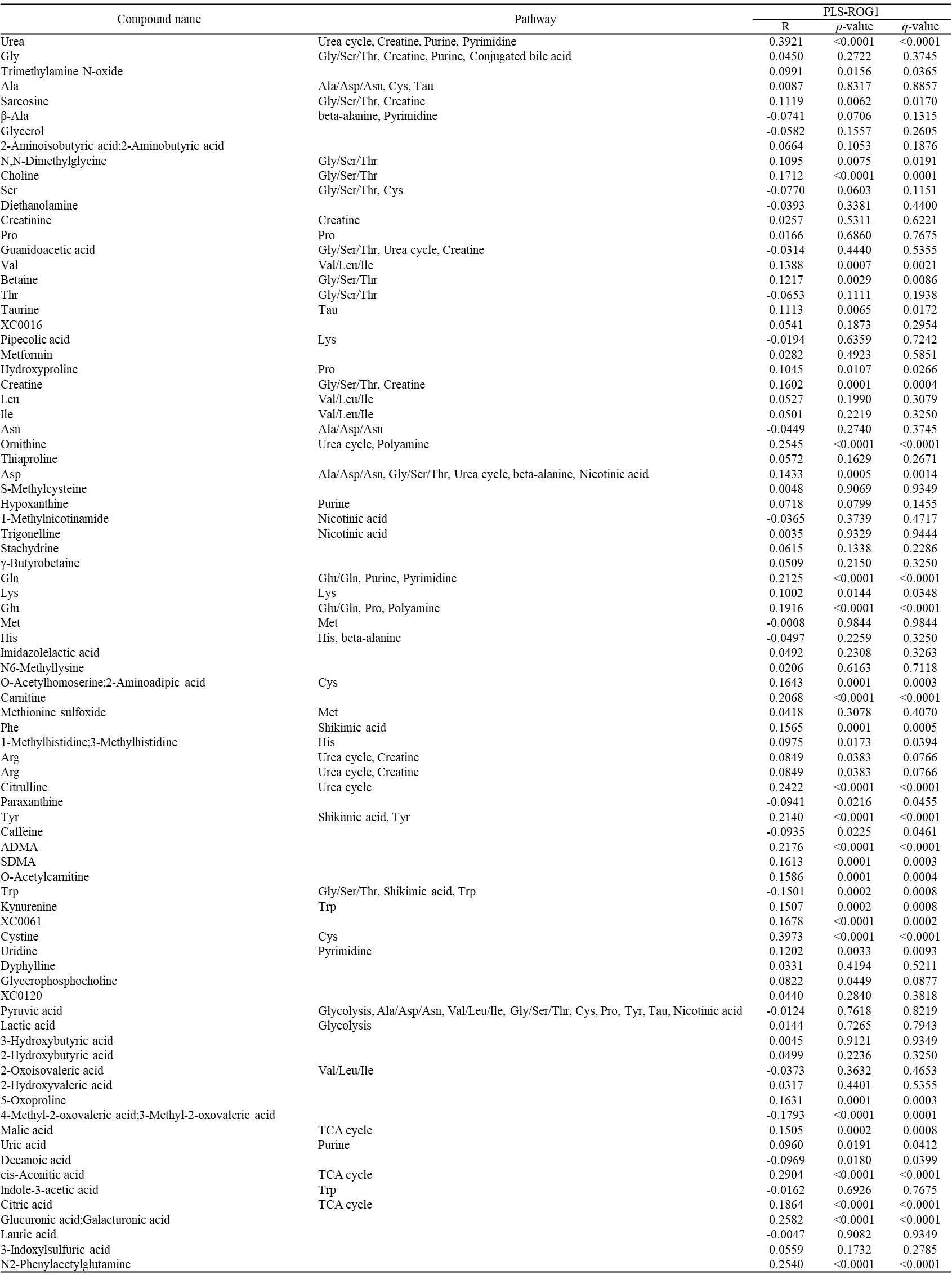
**Supplementary Table 1. Results of the first PLS-ROG loading in correlation with the Kellgren-Lawrence grade.**

S**upplementary Table 2. Metabolite set enrichment analysis to detect metabolic pathway with severity of knee osteoarthritis.**

| **Pathway** | **All** | **Detected** | **Selected** | **p-value** | **q-value** |
| --- | --- | --- | --- | --- | --- |
| **Enrichment from positively correlated metabolites** | | | | | |
| Urea cycle | 23 | 6 | 5 | 0.059 | 1.000 |
| TCA cycle | 13 | 3 | 3 | 0.085 | 1.000 |
| Glu/Gln | 11 | 2 | 2 | 0.196 | 1.000 |
| Polyamine | 20 | 2 | 2 | 0.196 | 1.000 |
| Pyrimidine | 56 | 4 | 3 | 0.230 | 1.000 |
| Creatine | 11 | 7 | 4 | 0.378 | 1.000 |
| Purine | 87 | 5 | 3 | 0.397 | 1.000 |
| Shikimic acid | 27 | 3 | 2 | 0.418 | 1.000 |
| Gly/Ser/Thr | 42 | 12 | 6 | 0.459 | 1.000 |
| Pro | 22 | 4 | 2 | 0.606 | 1.000 |
| Lys | 55 | 2 | 1 | 0.696 | 1.000 |
| His | 39 | 2 | 1 | 0.696 | 1.000 |
| Tyr | 76 | 2 | 1 | 0.696 | 1.000 |
| Cys | 30 | 5 | 2 | 0.745 | 1.000 |
| Trp | 80 | 3 | 1 | 0.835 | 1.000 |
| beta-alanine | 31 | 3 | 1 | 0.835 | 1.000 |
| Tau | 20 | 3 | 1 | 0.835 | 1.000 |
| Ala/Asp/Asn | 11 | 4 | 1 | 0.911 | 1.000 |
| Nicotinic acid | 43 | 4 | 1 | 0.911 | 1.000 |
| Val/Leu/Ile | 22 | 5 | 1 | 0.953 | 1.000 |
| **Enrichment from negatively correlated metabolites** | | | | | |
| Trp | 80 | 3 | 1 | 0.172 | 1.000 |
| Shikimic acid | 27 | 3 | 1 | 0.172 | 1.000 |
| Gly/Ser/Thr | 42 | 12 | 1 | 0.552 | 1.000 |

“All” includes the number of metabolites registered in metabolic pathway list. “Detected” includes the number of detected metabolites. “Selected” includes statistically significant metabolites with a p < 0.05. The p-value was computed by Fisher’s exact test and the q-value is its multiple comparison correction by Benjamini-Hochberg method.

**Supplementary Table 3. Demographic data of participants according to their effusion-synovitis score.**

|  | Effusion-synovitis score | | | | p-value |
| --- | --- | --- | --- | --- | --- |
|  | 0 | 1 | 2 | 3 |  |
| Number | 205 | 74 | 14 | 5 |  |
| Age | 55.4 ± 10.0 | 55.3 ± 10.0 | 61.3 ± 7.5 | 53.0 ± 5.9 | 0.155 |
| BMI | 22.0 ± 3.3 | 22.5 ± 3.4 | 23.0 ± 2.3 | 22.5 ± 3.2 | 0.171 |
| Muscle volume (kg) | 6.1 ± 0.7 | 6.3 ± 0.7 | 6.1 ± 0.8 | 6.3 ± 1.0 | 0.226 |
| Effusion volume (cm^3^) | 1.4 ± 0.6 | 3.7 ± 1.2 | 7.9 ± 1.5 | 17.0 ± 4.6 | <0.001 |

Data are presented as the mean ± standard deviation. Groups are compared by analysis of variance and Tukey test. BMI: body mass index.
